# Rationale and protocol for the Assessment of Impact of Real-time Continuous Glucose Monitoring on people presenting with severe Hypoglycaemia (AIR-CGM) study

**DOI:** 10.1186/s12902-019-0439-3

**Published:** 2019-10-26

**Authors:** Parizad Avari, Rozana Ramli, Monika Reddy, Nick Oliver, Rachael Fothergill

**Affiliations:** 1 Division of Diabetes, Endocrinology and Metabolism, Faculty of Medicine, Imperial College, 7S7a, Commonwealth Building, Hammersmith Campus, Du Cane Road, W12 0HS, London, UK; 2 Clinical Audit & Research Unit, London Ambulance Service NHS Trust, London, UK

**Keywords:** Type 1 diabetes, Continuous glucose monitoring, Severe hypoglycaemia

## Abstract

**Background:**

Severe hypoglycaemia carries a significant risk of morbidity and mortality for people with type 1 diabetes. Economic costs are also high, estimated at approximately £13 million annually in England, UK. Continuous glucose monitoring (CGM) has been shown to reduce hypoglycaemia and associated fear, improve overall glycaemia and quality of life, and is cost-effective. Despite effective pathways in place with high levels of resource utilization, it has been reported there are low levels of follow-up, therapy change and specialist intervention after severe hypoglycaemia. This study is designed to assess the impact of providing real-time CGM to people with type 1 diabetes, who have had a recent episode of severe hypoglycaemia (within 72 h), compared to standard care.

**Methods/design:**

Fifty-five participants with type 1 diabetes and a recent episode of severe hypoglycaemia, who are CGM naïve, will be recruited to the study. Participants will be randomised to CGM or standard care. The primary outcome is percentage time spent in hypoglycaemia (< 3.0 mmol/L, 55 mg/dL). Secondary outcomes include other measures of hypoglycaemia, time in euglycaemia, overall glucose status and patient reported qualitative measures.

**Discussion:**

This study assesses the impact of providing continuous glucose monitoring at the outset in individuals at highest risk of hypoglycaemia. Changing demand means that novel approaches need to be taken to healthcare provision. This study has the potential to shape future national standards.

**Trial registration:**

NCT03748433, November 2018 (UK).

## Background

Intensive glucose management with insulin therapy for people with Type 1 diabetes reduces the risk of microvascular complications and cardiovascular disease [1]. However, hypoglycaemia is a common iatrogenic complication that limits individuals from safely and effectively achieving their glycaemic goals. Repeated episodes of hypoglycaemia can substantially increase the risk of severe hypoglycaemia and are associated with increased frequency and severity of moderate hypoglycaemia [2, 3] and impaired awareness of hypoglycaemia [4]. Moreover, a preceding episode of severe hypoglycaemia is a powerful predictor of subsequent episodes of hypoglycaemia, independent of treatment intensity [5].

In adults, severe hypoglycaemia is defined as any episode of hypoglycaemia requiring the assistance of a third party to actively administer carbohydrate, glucagon, or take other corrective actions. On average, people with Type 1 diabetes report 1.8 self-treated incidences of hypoglycaemia per week, and 0.2–3.2 episodes of severe hypoglycaemia annually [6, 7]. This may be an underestimate.

Severe hypoglycaemia is associated with significant morbidity and even mortality, provoking major vascular events and causing neurological disability [1]. Between 4 and 10% of deaths in people with type 1 diabetes are attributed to hypoglycaemia [8] and the risk of severe hypoglycaemia increases 6-fold in people with impaired awareness of hypoglycaemia [9].

The impact of hypoglycaemia on health systems is widespread and includes both acute and chronic complications. In the UK diabetes consumes more than 10% of the National Health Service (NHS) budget [10] and in the USA a relatively greater amount is spent on type 1 compared with type 2 diabetes (8.6% of the diabetes budget compared with 5.6% of diabetes prevalence) [11].

The mean costs per hospital admission for hypoglycaemia in England is estimated to be in excess of £1000, with a total direct cost of severe hypoglycaemic episodes of around £13million each year [12–14]. Although hospital admissions for hypoglycaemia represent a small proportion of emergency department visits, they have substantial resource implications [15].

To address hypoglycaemia risk, regular self-monitoring of blood glucose up to 4–10 times daily and structured education, such as DAFNE (Dose Adjustment for Normal Eating) programme, are advocated in the National Institute of Clinical Excellence (NICE) guidance [16]. Despite such developments, severe hypoglycaemia remains a major hazard.

Continuous glucose monitoring (CGM) devices display an estimate of blood glucose levels, with alerts and alarms for impending and established hypo- and hyperglycaemia. In type 1 diabetes, real-time CGM has been shown to reduce hypoglycaemia [12, 17, 18], and improve overall glycaemia in all age groups when used continuously. Furthermore, CGM is associated with improvements in quality of life [19], reduced hypoglycaemia fear [20], and is cost-effective [21].

In the UK, CGM is supported by NICE for people with type 1 diabetes who are willing to commit to using CGM at least 70% of the time and who have any of the following despite optimised use of insulin therapy and conventional blood glucose monitoring [22]:

- More than 1 episode a year of severe hypoglycaemia with no obvious preventable precipitating cause.
- Complete loss of awareness of hypoglycaemia.
- Frequent (more than 2 episodes a week) asymptomatic hypoglycaemia that is causing problems with daily activities.
- Extreme fear of hypoglycaemia.
- Hyperglycaemia (HbA1c level of 75 mmol/mol [9%] or higher) that persists despite testing at least 10 times a day.

Over the last two decades, with growing demands on the NHS, emergency medical and ambulance services have been required to redefine their role and configuration. Emergency departments are under increased pressure to discharge individuals from the emergency department directly, with review by specialist diabetes nurses or out-patient follow-up with the diabetes team.

Similar to the rest of the UK, the London Ambulance Service NHS Trust (LAS) have developed integrated care pathways for hypoglycaemia management [23]. The referral pathway enables people with known diabetes requiring assistance for a hypoglycaemic episode to be assessed and treated by LAS, before either being transferred to hospital, or referred to their general practitioner or local NHS specialist diabetes service.

Despite development of effective hypoglycaemia pathways within emergency departments and ambulance services to primary, community and secondary care services for early review [24–26], it has been reported that there are low levels of follow-up, therapy change and specialist input following an episode of hypoglycaemia [27]. This is in line with the clinical experience of the authors.

The aims of this study “Assessment of the Impact of Real-time Continuous Glucose Monitoring on People Presenting with Severe Hypoglycaemia (AIR-CGM)” is to assess the impact of real-time CGM in people with type 1 diabetes, who have had a recent episode of severe hypoglycaemia (within 72 h), compared to usual care. The International Hypoglycaemia Study Group recommend reporting hypoglycaemia in studies as < 3.0 mmol/l, and is therefore used as the basis for the primary study outcome [28].

This is a collaborative study with the London Ambulance Service and to our best knowledge the first study to evaluate the impact of initiating real-time CGM soon after an episode of severe hypoglycaemia requiring an ambulance call-out or admission to the Emergency Department.

## Methods/Design

### Design

**Fig. 1:**
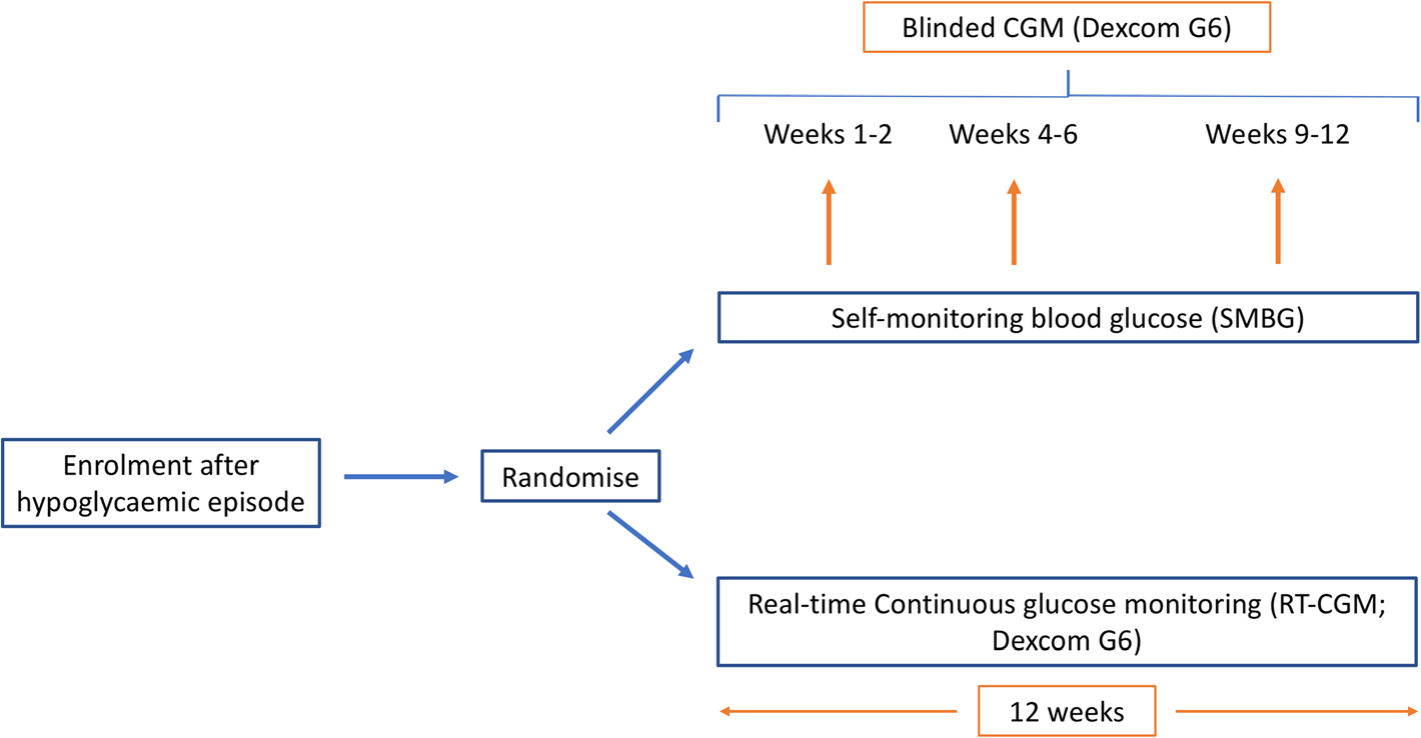
Study Design

This is a randomised, controlled trial comparing the impact of real-time CGM with usual care following severe hypoglycaemia (Fig. 1). Fifty-five participants will be followed up for 12 weeks.

### Eligibility - inclusion criteria

Participants are included if:

- Adults aged > 18 years;
- Episode of severe hypoglycaemia requiring ambulance call-out or emergency department attendance within 72 h;
- Type 1 diabetes confirmed on the basis of clinical features;
- Type 1 diabetes for greater than 3 years

### Eligibility - exclusion criteria

Participants are excluded if:

- CGM or flash glucose monitoring (Abbott Freestyle Libre device) used within the last 6 months (except for short periods of diagnostic blinded use under clinic supervision)
- Use of pre-mixed insulin;
- Pregnant or planning pregnancy;
- Breastfeeding;
- Enrolled in other clinical trials;
- Active malignancy or under investigation for malignancy;
- Reduced manual dexterity;
- Severe visual impairment;
- No access to smartphone or computer;
- Unable to participate due to other factors, as assessed by the Chief Investigators.

The aim of this study is to recruit people with type 1 diabetes at high-risk for recurrent severe hypoglycaemia who may benefit from CGM usage. The study population is in keeping with other clinical studies of adults at highest risk of hypoglycaemia (eg. HypoDE [29], IN CONTROL [30] and I-HART CGM [31]) and will include participants with long diabetes duration, hypoglycaemia-associated autonomic failure, as well as people experiencing their first episode of severe hypoglycaemia resulting from transient risk factors. People with enduring risks that may not be modified by CGM (such as drug or alcohol use) will be assessed on an individual basis and may be excluded with these data reported at the study end.

### Sample size and feasibility

London Ambulance Service NHS Trust (LAS) provides emergency services to a population of 8.6 million in London. The Ambulance service covers an area of approximately 620 mile^2^, supporting 39 Acute Trusts and 32 primary care Clinical Commissioning Groups (CCG). LAS attends approximately 20,000 diabetes related emergencies per year [23]. In 2015, the LAS recorded 2152 emergency calls for hypoglycaemia within North West London only (i.e. Harrow, Hillingdon, Brent, North Central, Hounslow and Hammersmith/Fulham). Of these, 64% of callouts resulted in conveyance to the Emergency Department. Furthermore, between 1st of April 2017 and 31st of March 2018 the LAS received 10,490 emergency calls for hypoglycaemia across Greater London, with 58.78% of these incidents conveyed to Emergency Departments.

In terms of attendances to the emergency departments, between 1st March 2014 and 28th February 2015, there were 236 attendance episodes at Imperial College Healthcare NHS Trust, with a coded diagnosis of hypoglycaemia.

Based on our previous findings from technology trials, we estimate the percentage time spent in hypoglycaemia (< 3.0 mmol/l) to be 64% lower in the CGM group compared to the self-monitoring group [mean (SD): CGM group 2.1% (2.3%); self-monitoring group 5.8% (5.9%)]. To demonstrate that difference as significant at *p* < 0.05 and with 80% power, 25 participants would be needed in each group. To allow for a 10% drop-out 55 participants will be recruited.

## Recruitment

**Fig. 2:**
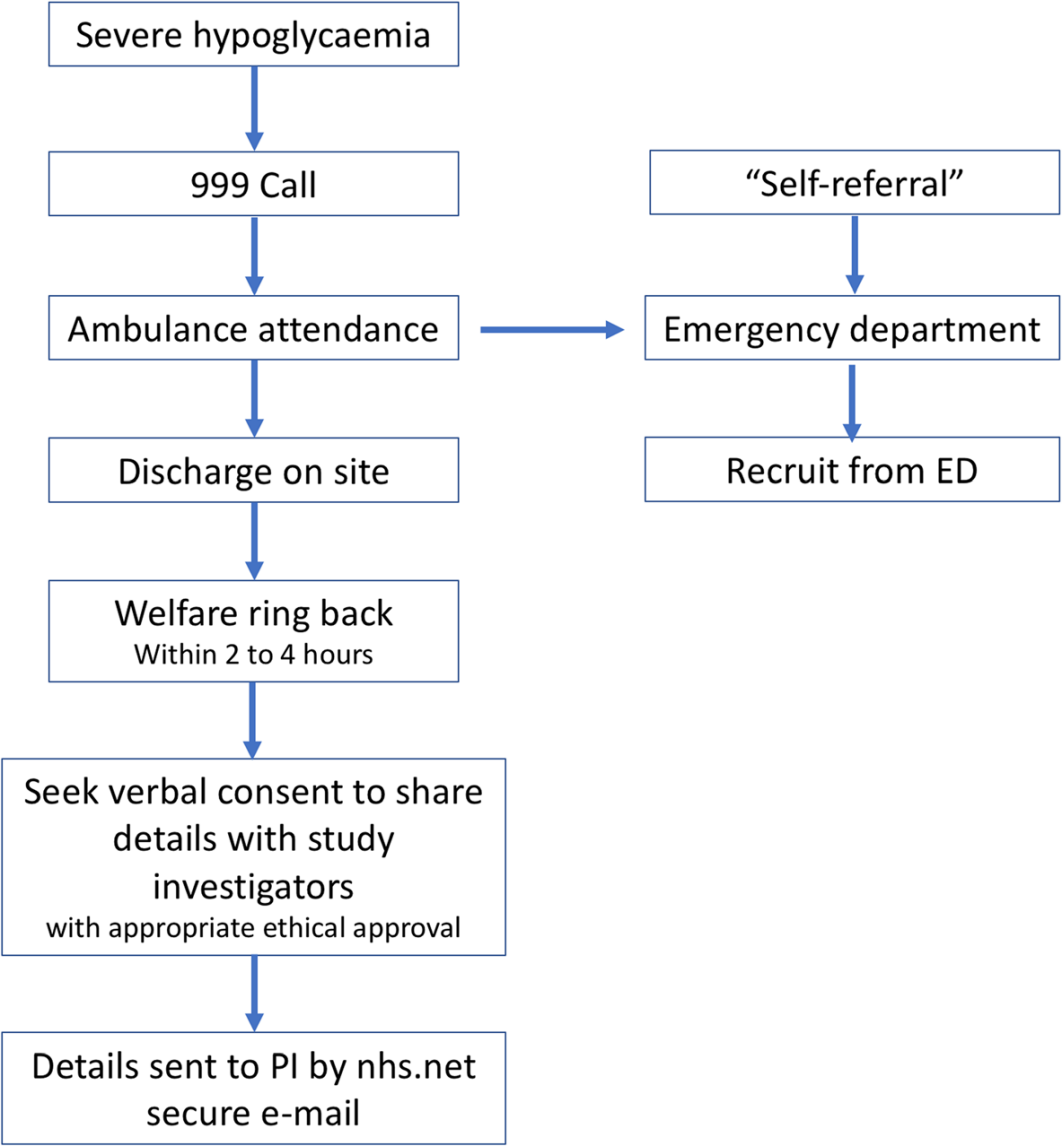
Recruitment Flow Chart

Recruitment is undertaken in collaboration with the LAS and the Emergency Departments of local NHS hospitals serving a diverse population in London (St Mary’s Hospital and Charing Cross Hospital; Fig. 2). LAS’s Referral Support Team will undertake pre-screening eligibility checks of individuals who have experienced severe hypoglycaemia and recover well enough following treatment by LAS clinicians to be left on scene (not transported to hospital). If the patient meets the study inclusion criteria, their details will be passed to a clinician in the Emergency Operations Centre who will contact the person within 2 h by telephone to check on their welfare. If the person has capacity and is well, the clinician will introduce the study and gain verbal consent to pass their name and telephone number, securely, to the research team (Fig. 2).

Potential participants will be identified from the Emergency Departments on a daily retrospective database search on admissions using the diagnosis code “hypoglycaemia”.

### Screening visit and enrolment

Following informed consent, study participants will give a full medical and medication history, as well as undergo a physical examination and ECG. Venous blood will be taken at 9 am for measurement of HbA1c, plasma glucose, renal function, serum C-peptide, thyroid function test, 9 am cortisol and coeliac screen (tissue transglutaminase antibody). A random urine sample will also taken for measurement of albumin:creatinine ratio, and women of childbearing age will have a urine pregnancy test to exclude pregnancy. If participants meet the inclusion criteria, they will be enrolled on to the study and complete questionnaires (Diabetes treatment satisfaction questionnaire [DTSQ], Gold Score, CGM usability, Problem Areas in Diabetes [PAID], and Fear of hypoglycaemia survey score [HFS-2]). Potential participants will be excluded if adrenal insufficiency is identified and will undergo appropriate investigation and treatment to address this. Additionally, if thyroid function is overtly abnormal or untreated coeliac disease is identified, as this may contribute to their ongoing potential for hypoglycaemia, the participant will be withdrawn.

Enrolled participants will be randomised to real-time CGM or self-monitoring blood glucose (SMBG) group. Randomisation to be performed using sealedenvelope.com and stratified by insulin delivery modality.

The real-time CGM group will receive Dexcom G6 transmitter and sensors, as well as a structured education refresher focusing on hypoglycaemia avoidance, recognition, and management. Participants in the SMBG group will receive the same structured education refresher focusing on hypoglycaemia avoidance, recognition, and management. All education is delivered from a predefined curriculum and is supported by independent written materials.

Participants will be instructed to test their capillary blood glucose if symptoms of hypo- or hyperglycaemia occur, in case of sensor failure or if the sensor glucose is out of the desired range (3.9 mmol/L – 13.3 mmol/L, 70 mg/dL – 240 mg/dL). Sensor change for the Dexcom G6 is required every 10 days as per manufacturer’s guidance (or sooner in event of sensor failure). Low glucose alert settings are standardised at 4.4 mmol/L (80 mg/dL) for all participants at the start of the study and may be reduced to 4.0 mmol/L (70 mg/dL) at week 2 during the telephone visit depending on participant preference. High glucose alerts can be personalised.

The SMBG group will additionally undergo blinded CGM at weeks 1 and 2, weeks 4 to 6 and weeks 9 to 12 using the Dexcom G6 system. Participants in this group will be shown how to insert the Dexcom G6 at the first clinic visit and sensors are provided so they can do this at home.

### Follow-up visits

Participants will be provided telephone support twice in the first week, then weekly for the next 3 weeks, and every 2 weeks thereafter, to optimise glucose and reduce the risk of further hypoglycaemia.

Participants in the CGM group with an iOS or Android smartphone will be able to upload and share data with the Research team through the manufacturer’s software. Participants who do not have a smartphone can upload their CGM data using a PC or Mac desktop computer with their CGM receivers. In total, participants will use CGM for 12 weeks.

Participants in the SMBG group will be asked to self-monitor a minimum of 4 times daily and upload data using appropriate software. They will receive telephone support from the research team, based on their SMBG data, at the same time points as the CGM group, to optimise glucose and reduce the risk of further hypoglycaemia. Blinded CGM data from the SMBG group will be uploaded at the end of the study.

### End of study

At the end of the study (i.e. at end of 12 weeks), repeat blood tests to assess HbA1c will be performed and questionnaires similar to that at baseline will be completed. Study equipment is returned, and individuals will resume to their standard care. Table 1 outlines a summary of participant visits.

**Table 1:**
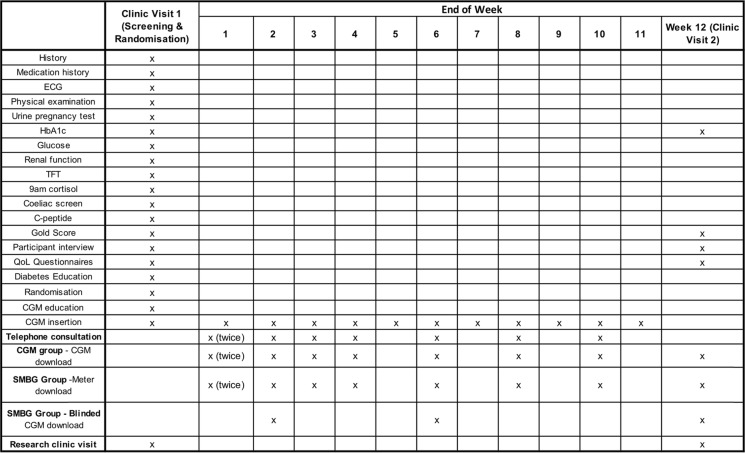
Summary table of investigations, treatment and assessments

## Study Outcomes

### Primary outcome

The primary outcome is percentage time spent in hypoglycaemia (< 3.0 mmol/L, 55 mg/dL).

### Secondary outcomes

- Number of episode of serious hypoglycaemia (defined as a sensor glucose < 3.0 mmol/L (55 mg/dL) for > 20 min)
- Number of episodes of severe hypoglycaemia requiring third party assistance
- Percentage time spent in hypoglycaemia (< 3.9 mmol/L, 70 mg/dL)
- Percentage time in euglycaemia (3.9–7.8 mmol/L, 70-140 mg/dL)
- Percentage time spent in target (3.9-10 mmol/L, 70-180 mg/dL)
- Percentage time spent in hyperglycaemia (> 10 mmol/L, 180 mg/dL)
- Number hypoglycaemic excursions
- Glucose variability
- HbA1c
- Ambulance call-out rates
- Summary measure of quality of life from questionnaires (DTSQ, CGM usability, PAID, HFS2)
- Gold score
- Cost effectiveness

### Ethics, informed consent and safety

The study is conducted in accordance with the recommendations for physicians involved in research on human subjects adopted by the 18th World Medical Assembly, Helsinki 1964 and later revisions.

Written consent is obtained from each participant by the research team after full explanation, an information leaflet offered, and time allowed for consideration. The right of the participant to refuse to participate, or continue participating, without giving reasons and without prejudicing further treatment is respected. The Chief Investigator ensures that participant confidentiality is respected, and local data protection requirements are met, in line with the Data Protection Act 2018.

Only research staff trained in Good Clinical Practice participate in the project to obtain informed consent and conduct procedures. The study is subject to inspection and audit by the sponsors, ensuring adherence to Good Clinical Practice and other aspects of research governance.

All adverse events (serious and non-serious) will be reported to the Chief Investigator in the first instance.

### Data

Data are locally collected and transferred to the central database. The data are coded and anonymised and then stored in an encrypted folder in a password protected computer.

### Publication policy

The results arising from this project will be disseminated by peer reviewed scientific journals, internal report, conference presentation and publication on websites. No identifiable personal data will be published.

All participants will be informed of the results at the conclusion of the study and details of any publications that arise from the study will be disseminated to participants.

### Statistics and data analysis

Analyses will be performed using Stata version 14.2. Baseline data will be taken from the first 14 days of monitoring and outcomes will be calculated from the last 30 days of the treatment period.

Analysis is based on an intention to treat principle. The primary and secondary outcomes will be assessed between the two study arms using a Wilcoxon Rank sum test. Other outcomes assessed will include hypoglycaemia awareness by participant (Gold score). Hypoglycaemia outcomes will be assessed throughout the time period and nocturnally (22:00–06:00 h).

## Discussion

This study is the first to evaluate the impact of real-time CGM initiation, compared to usual care, following an episode of severe hypoglycaemia requiring an ambulance call-out or admission to the Emergency Department.

Addressing severe hypoglycaemia to reduce the risk of further episodes and acting promptly to optimise hypoglycaemia awareness is critical in people at high risk. If the results of this study are positive, enabling individuals to have direct access to CGM in a time-effective manner following severe hypoglycaemia, may provide an effective adjunct to diabetes self-management with improved glycaemia, greater treatment satisfaction and associated reduction in costs to the National Health Service and Ambulance services.

## Data Availability

Not applicable

## Abbreviations

CCG: Clinical Commissioning Group
CGM: Continuous Glucose Monitoring
DAFNE: Dose Adjustment for Normal Eating
DTSQ: Diabetes treatment satisfaction questionnaire
HFS-2: Fear of hypoglycaemia survey score
LAS: London Ambulance Service
NHS: National Health Service
NICE: National Institute of Clinical Excellence
PAID: Problem Areas in Diabetes
SD: Standard deviation
SMBG: Self-monitoring blood glucose
UK: United Kingdom
USA: United States of America
